# Crystal structure and Hirshfeld surface analysis of (*E*)-*N*-(2-styrylphen­yl)benzene­sulfonamide

**DOI:** 10.1107/S2056989024008892

**Published:** 2024-09-20

**Authors:** Dharani Albert A. M., Achyuta Nagaraj, Kanagasabai Somarathinam, Pavunkumar Vinayagam, Mohanakrishnan Arasambattu K., Gugan Kothandan

**Affiliations:** a CAS in Crystallography and Biophysics University of Madras,Chennai India; b Department of Organic Chemistry University of Madras,Chennai India; Institute of Chemistry, Chinese Academy of Sciences

**Keywords:** crystal structure, synthesis, benzene­sulfonamide, hydrogen bonding, Hirshfeld surface analysis, docking, EGFR kinase

## Abstract

The crystal structure of the title compound C_20_H_17_NO_2_S features hydrogen-bonding and C—H⋯π inter­actions. Hirshfeld surface analysis revealed that H⋯H, C⋯H/H⋯C and O⋯H/H⋯O inter­actions make a major contribution to the crystal packing. Docking studies were carried out to determine the binding affinity and inter­action profile of the title compound with EGFR kinase, a member of the ErbB family of receptor tyrosine kinases, which is crucial for processes such as cell proliferation and differentiation. The title compound shows a strong binding affinity with EGFR kinase, with the most favourable conformation having a binding energy of −8.27 kcal mol^−1^ and a predicted IC50 of 870.34 n*M*, indicating its potential as a promising candidate for targeted lung cancer therapy.

## Abstract 2

The crystal structure of the title compound C_20_H_17_NO_2_S features hydrogen-bonding and C—H⋯π. Docking studies show that it has a strong binding affinity with EGFR kinase, indicating its potential as a promising candidate for targeted lung cancer therapy.

## 1. Chemical context

The indole structure is widely regarded as a privileged scaffold, capable of serving as a ligand for various biological targets (Kaushik *et al.*, 2013 ▸). Indoles are prevalent across a broad spectrum of natural sources, including plants, animals and microorganisms. Numerous indole-containing compounds exhibit notable biological activities; for instance, indole-based alkaloids such as serotonin, tryptamine, and ergotamine are crucial in regulating physiological processes and significantly impact human health and behaviour. Indoles are also present in a variety of pharmaceuticals, such as anti­psychotic, anti­depressant and anti­microbial drugs. Beyond their biological significance, indoles are valuable as they are versatile building blocks in organic synthesis, with the indole ring being functionalized and modified to produce a diverse array of chemical compounds. Although many methods for synthesizing indole derivatives exist, there remains a strong inter­est in developing new and more efficient synthesis techniques. The transformation of 2-alkenylanilines into indoles has gained popularity as a straightforward approach due to the widespread availability of both anilines and alkenes (or styrenes). One such method involves C—H amination *via* transition-metal catalysts. Recently, methods that avoid the use of metals in cyclization have garnered considerable attention (Hegedus *et al.*, 1978 ▸; Larock *et al.*, 1996 ▸; Maity *et al.*, 2012 ▸; Youn *et al.*, 2015 ▸, 2016 ▸). A reaction was carried out with the aim of synthesizing 2-phenyl­indole from (*E*)-*N*-(2-styrylphen­yl)benzene­sulfon­amide through PIDA/BF_3_·OEt_2_-mediated intra­molecular cyclization and the structure of the (*E*)-*N*-(2-styrylphen­yl)benzene­sulfonamide inter­mediate of 2-phenyl­indole was confirmed through X-ray diffraction analysis.
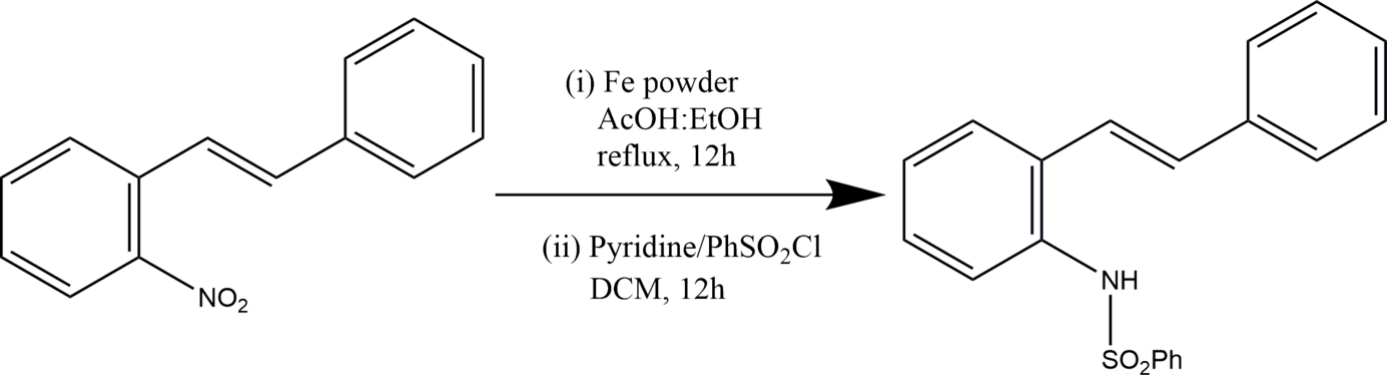


## 2. Structural commentary

**Figure 1:**
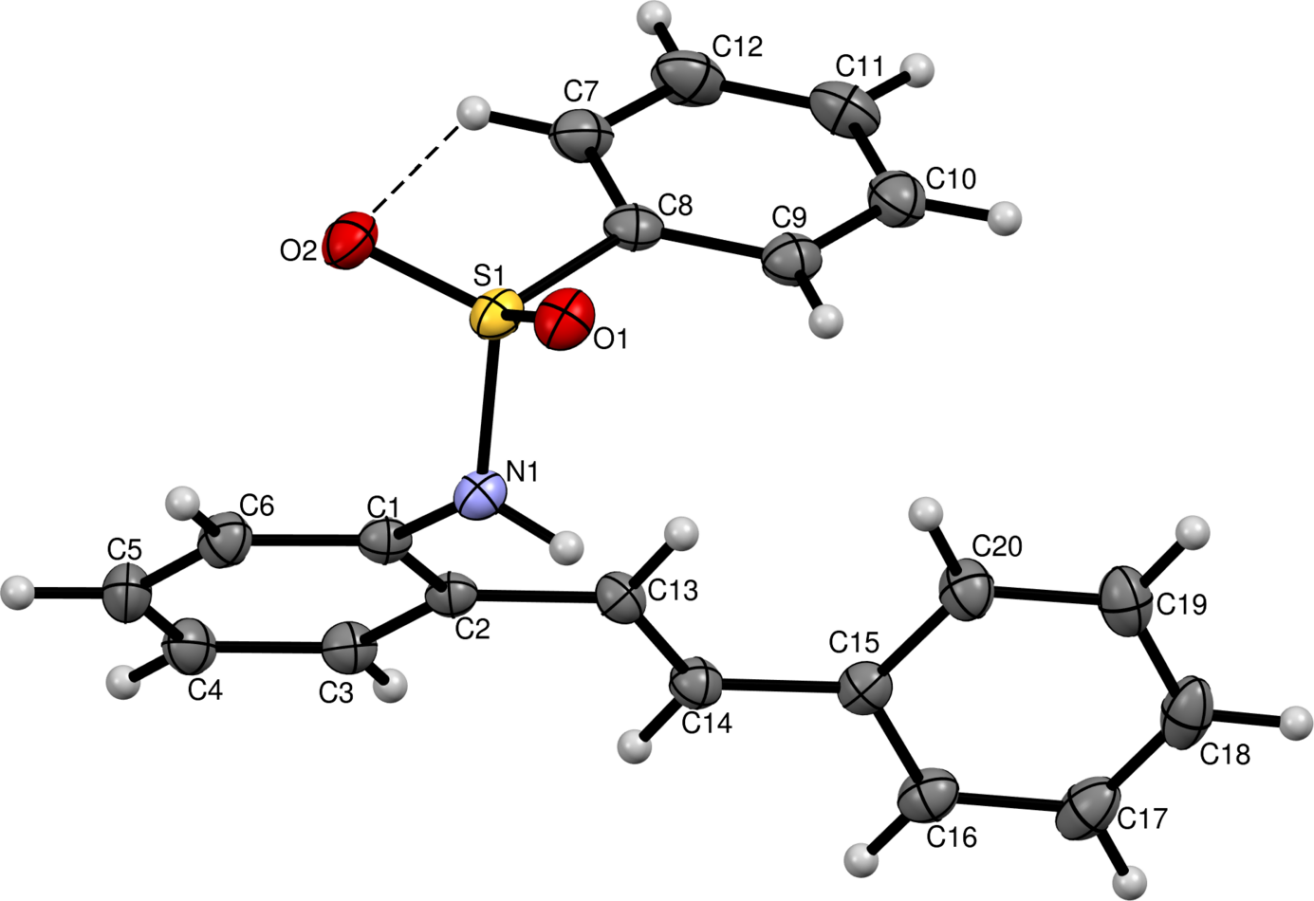
View of title compound showing the atom-numbering scheme with displacement ellipsoids drawn at the 50% probability level. The intra­molecular C7—H7⋯O2 hydrogen bond is shown as a dashed line.

In the title compound, the sulfur atom is bound to two oxygens, a nitro­gen (which is connected to another aromatic ring) and a carbon atom, forming a tetra­hedral structure between the two aromatic moieties with sulfur at the centre. Relevant bond lengths and angles are given in Table 1 ▸. For the C1–C6 ring, the weighted average bond distance is 1.3959 Å, the weighted average absolute torsion angle is 0.34° and the pseudo-rotation parameter (τ) is 0.3°. The C7–C12 ring has a weighted average bond distance of 1.3899 Å, a weighted average absolute torsion angle of 0.83° and a τ value of 0.8. Similarly, the C15–C20 ring exhibits a weighted average bond distance of 1.3925 Å, a weighted average absolute torsion angle of 1.76° and τ value of 1.8°. An intra­molecular C7—H7⋯O2 hydrogen bond (Fig. 1 ▸, Table 2 ▸) directs the relative orientation of the C7–C12 ring in the mol­ecular structure.

## 3. Supra­molecular features

**Figure 2:**
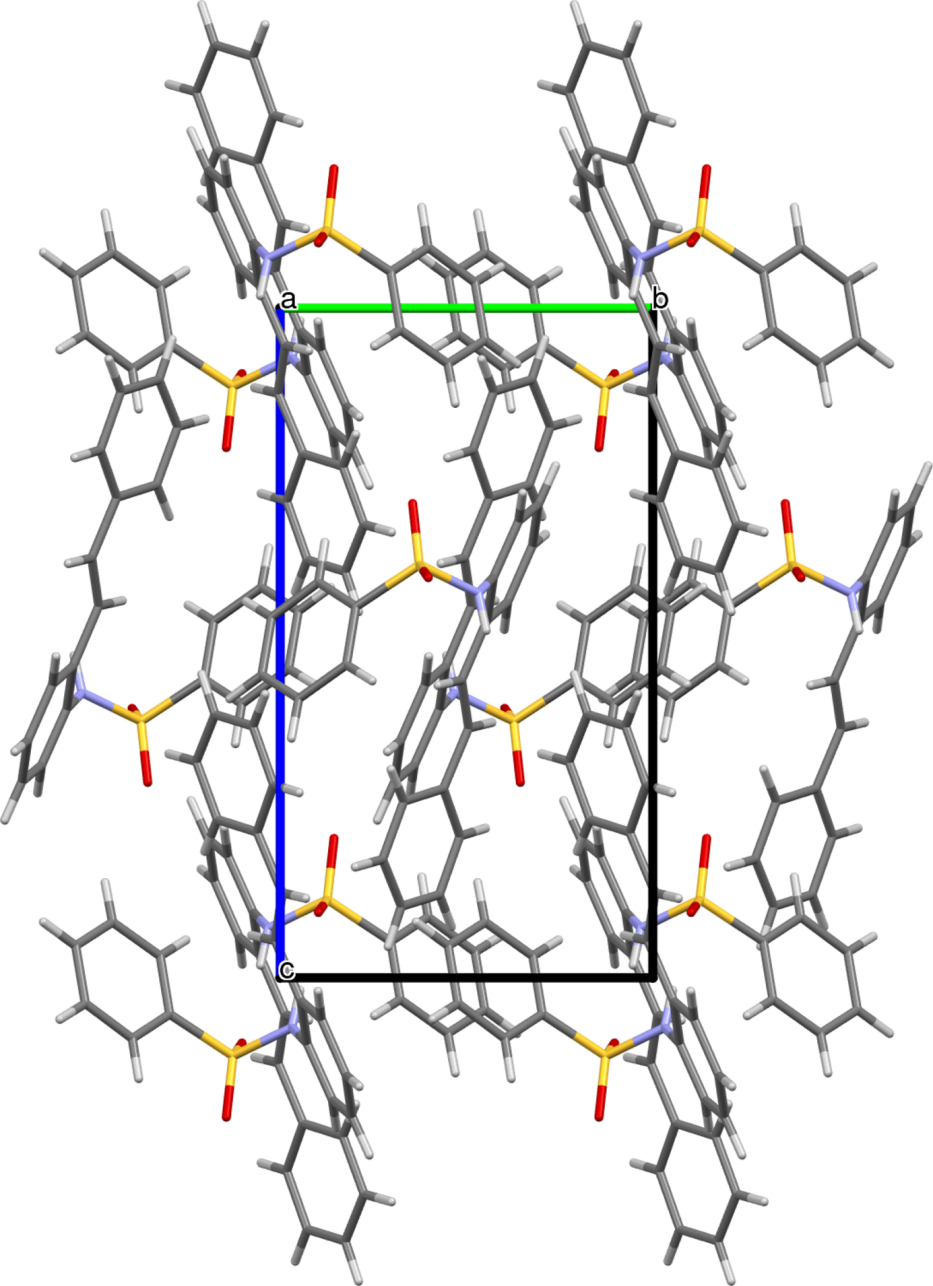
The crystal packing of the title compound viewed along the *a* axis.

In the crystal, N1—H1⋯O1 and C6—H6⋯O2 hydrogen bonds and C—H⋯π inter­actions (Table 1 ▸) are observed. The packing is shown in Fig. 2 ▸.

## 4. Database survey

A search in the Cambridge Structural Database (CSD, Version 5.45; Groom *et al.*, 2016 ▸) for the term ‘(styrylphen­yl)benzene­sulfonamide’ gave one hit, (*Z*)-*N*-(di­fluoro­meth­yl)-4-methyl-*N*-(2-styrylphen­yl)benzene­sulfonamide (CSD refcode HINBEO; Polley *et al.*, 2018 ▸). In this structure there is a difluromethyl group attached to the nitro­gen in addition to a methyl group at the *para* position of the benzene ring of benzene­sulfonamide.

## 5. Hirshfeld surface analysis

**Figure 3:**
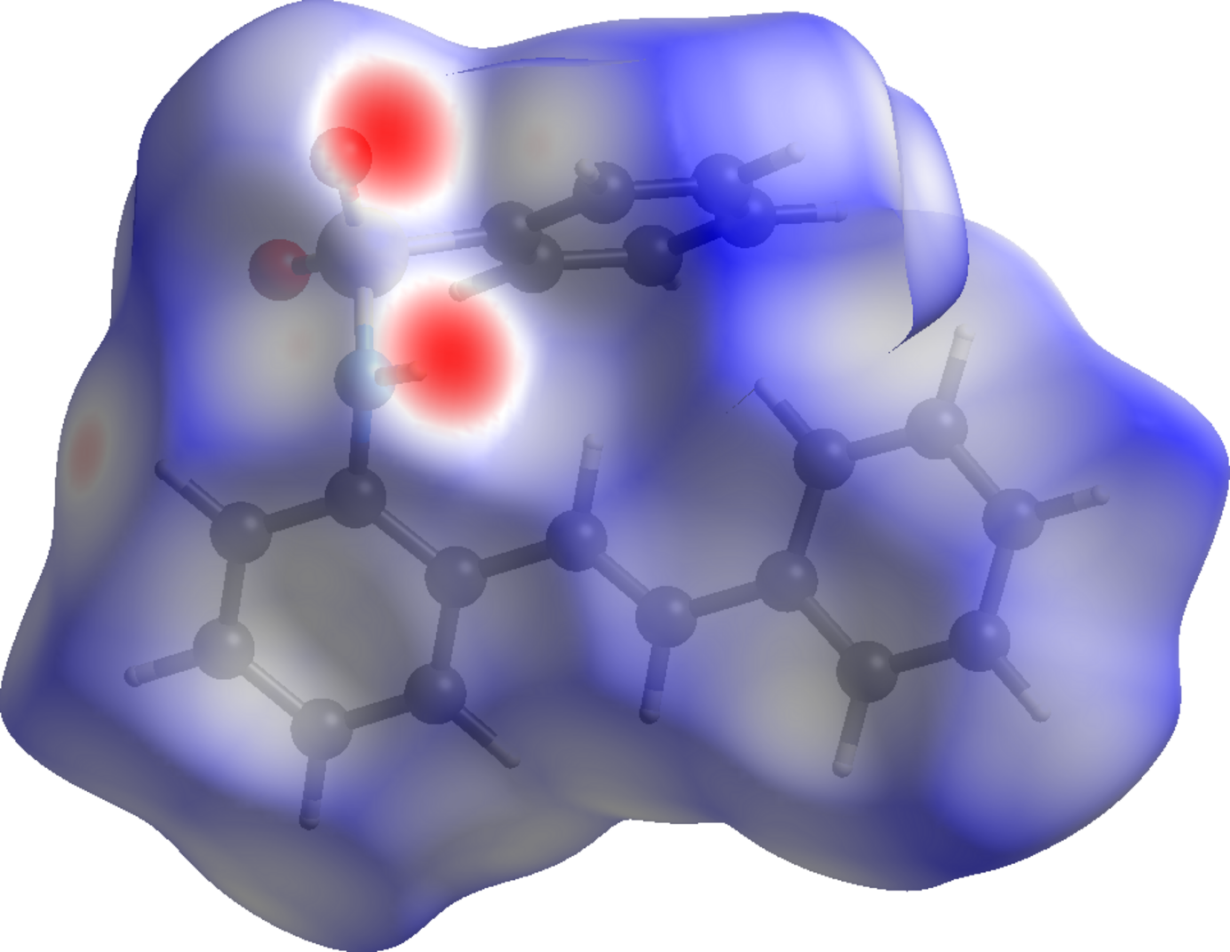
The Hirshfeld surface of the title compound mapped over *d*_norm_.

The Hirshfeld surface analysis was carried out using *Crystal Explorer 21* (Spackman *et al.*, 2021 ▸) to study the non-covalent inter­actions and the inter­atomic contacts. The Hirshfeld surface mapped over *d*_norm_ with shorter contacts in red, contacts around the van der Waals separation in white and longer contacts in blue is shown in Fig. 3 ▸.

**Figure 4:**
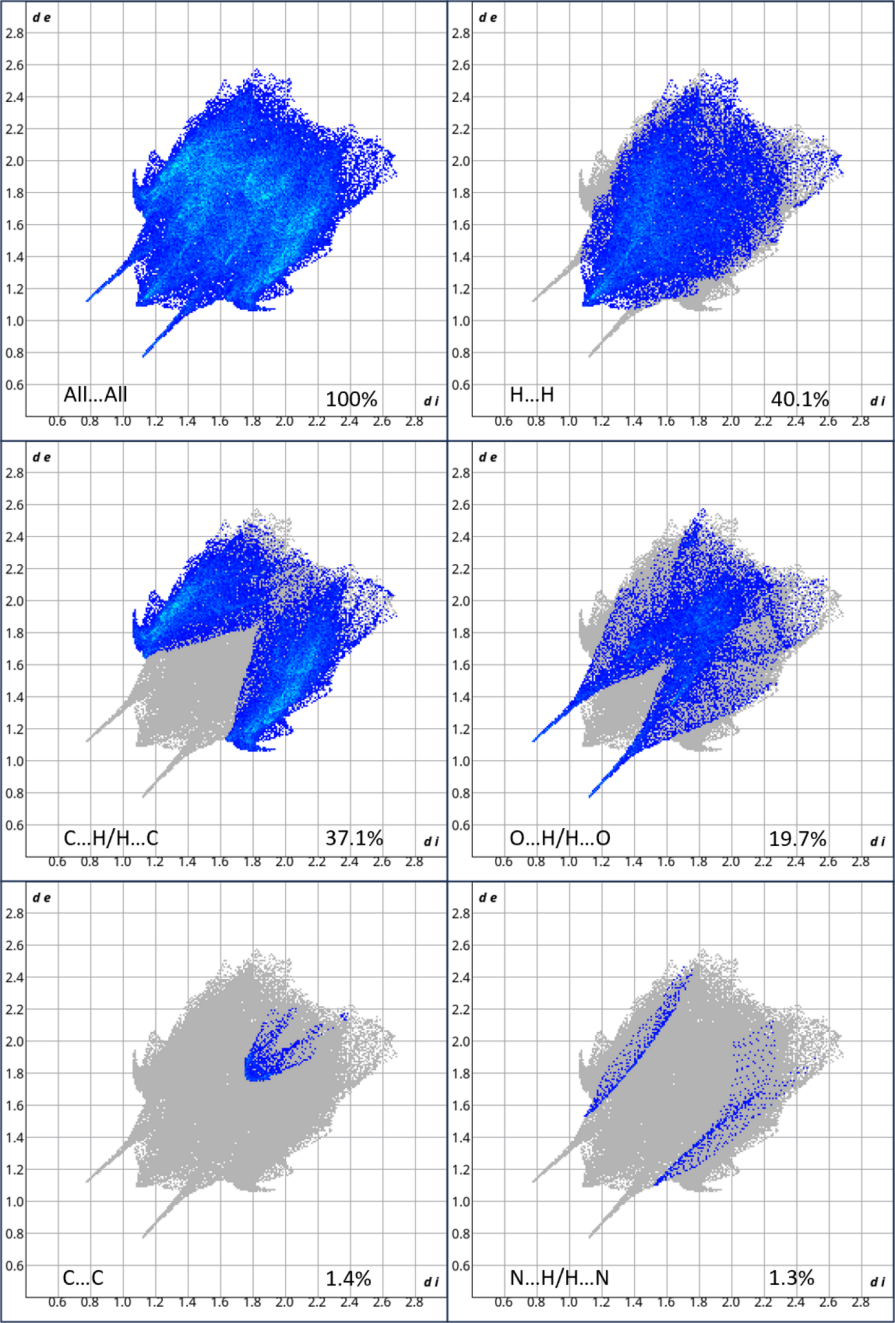
The various two dimensional fingerprint plots with the significant contacts labelled.

The two-dimensional fingerprint plots for significant contacts are given in Fig. 4 ▸. The contacts making the largest contributions are H⋯H (40.1%) due to the large number of hydrogen atoms in the mol­ecule, C⋯H/H⋯C (37.1%) and O⋯H/H⋯O (19.7%). Contacts making minor contributions include C⋯C (1.4%), N⋯H/H⋯N (1.3%) and O⋯C/C⋯O (0.4%).

## 6. *In silico* analysis

Mol­ecular docking studies were carried out to assess the potential of the title compound as a therapeutic agent by targeting EGFR kinase, a key protein involved in lung cancer development (Kavarthapu *et al.*, 2021 ▸). Dysregulation of EGFR, often through mutations or overexpression, is a major driver of non-small cell lung cancer (NSCLC), making it a key therapeutic target.

Docking was carried out using *AutoDock 4.2* (Morris *et al.*, 2009 ▸) software, with the EGFR kinase’s high-resolution 3D crystal structure (PDB ID: 2ITY; Yun *et al.*, 2007 ▸) obtained from the Protein Data Bank (Berman *et al.*, 2000 ▸). Prior to docking, co-crystallized ligands and solvent mol­ecules were removed using *PyMOL* (DeLano, 2002 ▸), the polar hydrogen atoms were added and the Kollman and Gasteiger charges were assigned to the protein. *AutoGrid* was used to calculate grid parameters, with a 40 × 40 × 40 point grid box and a spacing of 0.375 Å, centered on the binding site determined by the ligand-bound EGFR kinase (2ITY). Docking was conducted with the Lamarckian Genetic Algorithm (LGA) for 100 independent runs, keeping all other parameters at default. The protein was treated as rigid, while the ligand was allowed full flexibility. Docking results were evaluated based on binding inter­actions, binding energy (kcal mol^−1^), and predicted inhibitory concentration (IC50). The docking results showed that (*E*)-*N*-(2-styrylphen­yl)benzene­sulfonamide has a strong binding affinity for EGFR kinase, with the most favourable conformation having a binding energy of −8.27 kcal mol^−1^ and a predicted IC50 of 870.34 n*M*.

**Figure 5:**
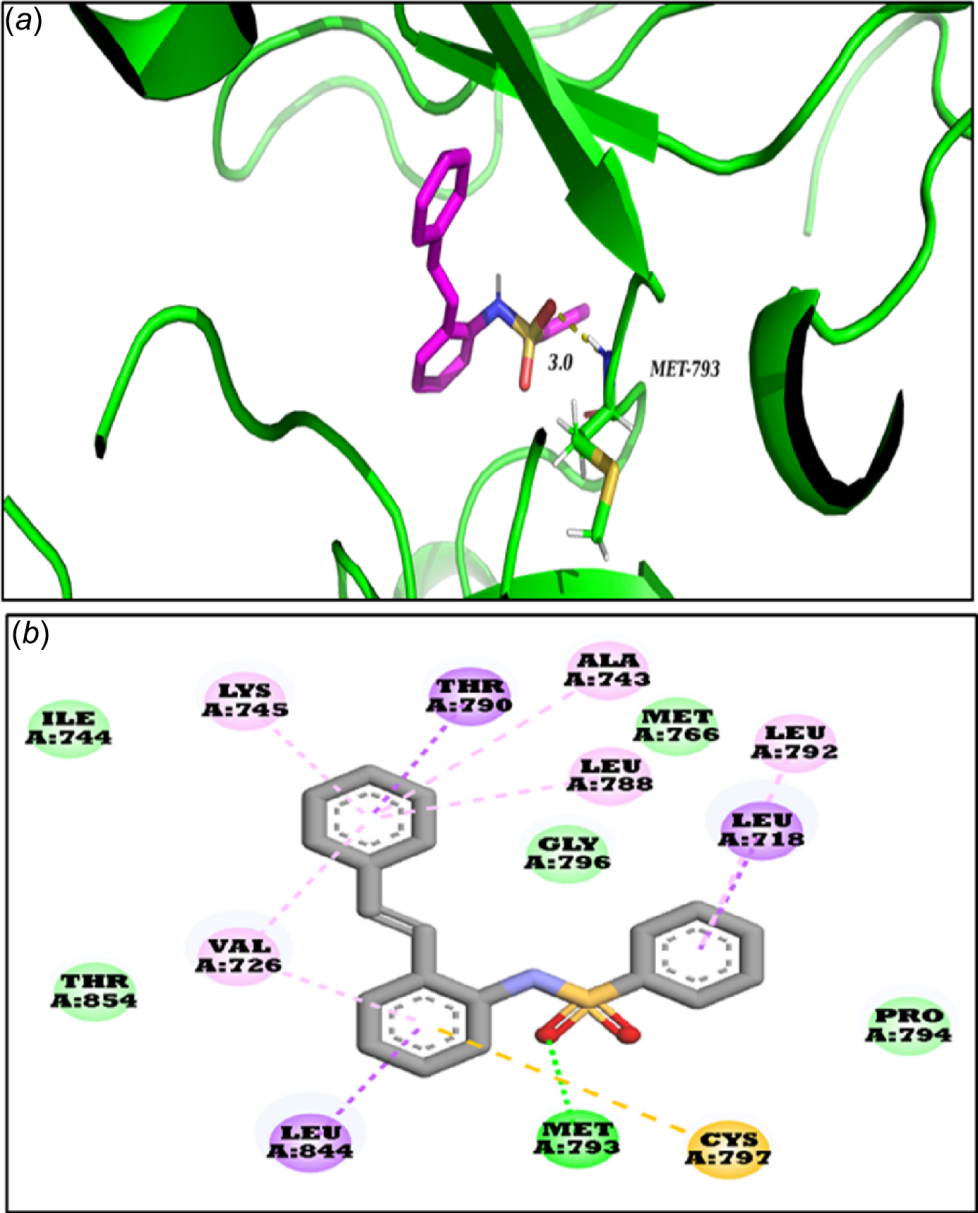
Mol­ecular docking results illustrating the inter­action of the title compound with EGFR kinase. (*a*) Hydrogen-bond inter­action and (*b*) overall inter­actions (the vdW, π–alkyl, π–sigma, and π–sulfur inter­actions are indicated in green, pink, purple, and yellow, respectively)

Further inter­action analysis shows that the ligand forms a hydrogen bond with the MET-793 residue at 3.0 Å, a crucial inter­action for the stability of the ligand–protein complex (Fig. 5 ▸). Additionally, the compound engages in various non-covalent inter­actions, including π–alkyl with VAL-726, ALA-743, LYS-745, LEU-788, and LEU-792; π–sigma with LEU-718, THR-790, and LEU-844; pi-sulfur with CYS-797; and van der Waals with ILE-744, MET-766, PRO-794, GLY-796, and THR-854. These inter­actions collectively enhance the ligand’s stability and affinity for EGFR kinase.

Considering EGFR’s critical role in NSCLC, the inter­action profile suggests the potential of the title compound as a therapeutic agent. Its strong binding affinity and specific inter­actions with EGFR kinase highlight its promise for further development in targeted lung cancer treatment, particularly for patients with EGFR mutations.

## 7. Synthesis and crystallization

To a hot solution of (*E*)-1-nitro-2-styryl­benzene (2.9 g, 12.88 mmol) in 50 mL of an EtOH–AcOH mixture (4:1 ratio), Fe powder (3.5 g, 64.40 mmol) was added, and the reaction mixture was refluxed for 6 h. Once the reaction was complete, as monitored by TLC, the solution was carefully deca­nted to remove the iron residue and then poured over crushed ice (100 g) containing 5 mL of concentrated HCl. The resulting solid was filtered and dried over CaCl_2_. The crude product was used directly in the next step without further purification. Subsequently, a solution of the resulting amine salts (2.2 g, 9.52 mmol) in dry DCM (20 mL) was prepared, to which benzene­sulfonyl chloride (1.3 mL, 10.47 mmol) and pyridine (0.92 mL, 11.42 mmol) were slowly added. The mixture was stirred at room temperature for 8 h under a nitro­gen atmosphere. After the reaction was complete, as monitored by TLC, the mixture was poured into ice–water (50 mL) containing 1 mL of concentrated HCl, extracted with DCM (2 × 20 mL), and then washed with water (2 × 20 mL) and dried over Na_2_SO_4_. The solvent was removed under reduced pressure, and the crude product was triturated with diethyl ether (10 mL), yielding (*E*)-*N*-(2-styrylphen­yl)benzene­sulfonamide (2.3 g, 61% yield over two steps) as a white solid, m.p. 399–401 K.

## 8. Refinement

Crystal data, data collection and structure refinement details are summarized in Table 3 ▸. The N-bound H atom was fully refined. C-bound H atoms were positioned geometrically (C—H = 0.95 Å) with *U*_iso_(H) = 1.2*U*_eq_(C).

## Supplementary Material

Crystal structure: contains datablock(s) I. DOI: 10.1107/S2056989024008892/nx2013sup1.cif

Structure factors: contains datablock(s) I. DOI: 10.1107/S2056989024008892/nx2013Isup2.hkl

CCDC reference: 2364967

Additional supporting information:  crystallographic information; 3D view; checkCIF report

## Figures and Tables

**Table 1:** Selected geometric parameters (Å, °)

S1—O1	1.4422 (9)	S1—C8	1.7653 (12)
S1—O2	1.4300 (9)	N1—C1	1.4343 (14)
S1—N1	1.6342 (10)		
			
O1—S1—N1	105.29 (5)	O2—S1—C8	108.68 (6)
O1—S1—C8	107.63 (6)	N1—S1—C8	106.87 (5)
O2—S1—O1	119.83 (5)	C1—N1—S1	121.88 (8)
O2—S1—N1	107.85 (5)		

**Table 2:** Hydrogen-bond geometry (Å, °) *Cg*1 is the centroid of the C1–C6 ring.

*D*—H⋯*A*	*D*—H	H⋯*A*	*D*⋯*A*	*D*—H⋯*A*
N1—H1⋯O1^i^	0.888 (18)	2.010 (18)	2.8907 (14)	170.8 (17)
C6—H6⋯O2^ii^	0.95	2.53	3.3332 (16)	143
C7—H7⋯O2	0.95	2.54	2.9208 (16)	104
C12—H12⋯*Cg*1^iii^	0.95	2.59	3.4333 (15)	148
C19—H19⋯*Cg*1^iv^	0.95	2.81	3.5747 (15)	141

Symmetry codes: (i) 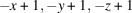; (ii) 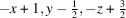; (iii) 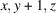; (iv) 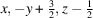.

**Table 3:** Experimental details

Crystal data	
Chemical formula	C_20_H_17_NO_2_S
*M*_r_	335.40
Crystal system, space group	Monoclinic, *P*2_1_/*c*
Temperature (K)	100
*a*, *b*, *c* (Å)	13.7320 (1), 8.2475 (1), 15.5387 (2)
β (°)	107.505 (1)
*V* (Å^3^)	1678.33 (3)
*Z*	4
Radiation type	Cu *K*α
μ (mm^−1^)	1.80
Crystal size (mm)	0.21 × 0.14 × 0.1

Data collection	
Diffractometer	SuperNova, Dual, Cu at home/near, HyPix
Absorption correction	Gaussian (*CrysAlis PRO*; Rigaku OD, 2022 ▸)
*T*_min_, *T*_max_	0.560, 1.000
No. of measured, independent and observed [*I* > 2σ(*I*)] reflections	37664, 3562, 3380
*R*_int_	0.039
(sin θ/λ)_max_ (Å^−1^)	0.634

Refinement	
*R*[*F*^2^ > 2σ(*F*^2^)], *wR*(*F*^2^), *S*	0.032, 0.084, 1.07
No. of reflections	3562
No. of parameters	221
H-atom treatment	H atoms treated by a mixture of independent and constrained refinement
Δρ_max_, Δρ_min_ (e Å^−3^)	0.36, −0.46

Computer programs: *CrysAlis PRO* (Rigaku OD, 2022 ▸), *SHELXT2018/2* (Sheldrick, 2015*a* ▸), *SHELXL2019/3* (Sheldrick, 2015*b* ▸) and *OLEX2* (Dolomanov *et al.*, 2009 ▸).

## supplementary crystallographic information

### N-{2-[(E)-2-Phenylethenyl]phenyl}benzenesulfonamide Crystal data

**Table:**

C_20_H_17_NO_2_S	*F*(000) = 704
*M**_r_* = 335.40	*D*_x_ = 1.327 Mg m^−^^3^
Monoclinic, *P*2_1_/*c*	Cu *K*α radiation, λ = 1.54184 Å
*a* = 13.7320 (1) Å	Cell parameters from 18551 reflections
*b* = 8.2475 (1) Å	θ = 5.1–77.6°
*c* = 15.5387 (2) Å	µ = 1.80 mm^−^^1^
β = 107.505 (1)°	*T* = 100 K
*V* = 1678.33 (3) Å^3^	Block, clear intense colourless
*Z* = 4	0.21 × 0.14 × 0.1 mm

### N-{2-[(E)-2-Phenylethenyl]phenyl}benzenesulfonamide Data collection

**Table:**

SuperNova, Dual, Cu at home/near, HyPix diffractometer	3562 independent reflections
Radiation source: micro-focus sealed X-ray tube, SuperNova (Cu) X-ray Source	3380 reflections with *I* > 2σ(*I*)
Mirror monochromator	*R*_int_ = 0.039
Detector resolution: 10.0000 pixels mm^-1^	θ_max_ = 77.7°, θ_min_ = 3.4°
ω scans	*h* = −17→17
Absorption correction: gaussian (CrysAlisPro; Rigaku OD, 2022)	*k* = −10→10
*T*_min_ = 0.560, *T*_max_ = 1.000	*l* = −19→19
37664 measured reflections	

### N-{2-[(E)-2-Phenylethenyl]phenyl}benzenesulfonamide Refinement

**Table:**

Refinement on *F*^2^	Primary atom site location: dual
Least-squares matrix: full	Hydrogen site location: mixed
*R*[*F*^2^ > 2σ(*F*^2^)] = 0.032	H atoms treated by a mixture of independent and constrained refinement
*wR*(*F*^2^) = 0.084	*w* = 1/[σ^2^(*F*_o_^2^) + (0.0413*P*)^2^ + 0.655*P*] where *P* = (*F*_o_^2^ + 2*F*_c_^2^)/3
*S* = 1.07	(Δ/σ)_max_ = 0.001
3562 reflections	Δρ_max_ = 0.36 e Å^−^^3^
221 parameters	Δρ_min_ = −0.46 e Å^−^^3^
0 restraints	

### N-{2-[(E)-2-Phenylethenyl]phenyl}benzenesulfonamide Special details

**Table:**

Geometry. All esds (except the esd in the dihedral angle between two l.s. planes) are estimated using the full covariance matrix. The cell esds are taken into account individually in the estimation of esds in distances, angles and torsion angles; correlations between esds in cell parameters are only used when they are defined by crystal symmetry. An approximate (isotropic) treatment of cell esds is used for estimating esds involving l.s. planes.

### N-{2-[(E)-2-Phenylethenyl]phenyl}benzenesulfonamide Fractional atomic coordinates and isotropic or equivalent isotropic displacement parameters (Å^2^)

**Table:**

	*x*	*y*	*z*	*U*_iso_*/*U*_eq_	
S1	0.47127 (2)	0.62654 (3)	0.61679 (2)	0.02047 (10)	
O1	0.56671 (6)	0.60255 (11)	0.59762 (6)	0.0253 (2)	
O2	0.47052 (7)	0.64449 (11)	0.70812 (6)	0.0267 (2)	
N1	0.40171 (7)	0.46841 (12)	0.57421 (7)	0.0195 (2)	
C1	0.31138 (8)	0.42782 (14)	0.59774 (8)	0.0187 (2)	
C2	0.21387 (8)	0.44410 (13)	0.53465 (8)	0.0183 (2)	
C6	0.32417 (10)	0.35980 (15)	0.68241 (8)	0.0235 (3)	
H6	0.390750	0.349266	0.723729	0.028*	
C3	0.13033 (9)	0.38948 (14)	0.56134 (8)	0.0212 (2)	
H3	0.063492	0.398483	0.520299	0.025*	
C15	0.10379 (9)	0.56186 (14)	0.28252 (8)	0.0211 (2)	
C13	0.20040 (8)	0.51323 (14)	0.44460 (8)	0.0197 (2)	
H13	0.254825	0.576531	0.436591	0.024*	
C14	0.11773 (9)	0.49428 (14)	0.37298 (8)	0.0214 (2)	
H14	0.063239	0.431619	0.381352	0.026*	
C8	0.41068 (9)	0.79664 (14)	0.55434 (8)	0.0217 (2)	
C9	0.41757 (9)	0.82218 (15)	0.46753 (9)	0.0243 (2)	
H9	0.458356	0.753136	0.443539	0.029*	
C20	0.17418 (10)	0.66848 (16)	0.26366 (9)	0.0260 (3)	
H20	0.232179	0.702399	0.311078	0.031*	
C16	0.01775 (9)	0.51760 (15)	0.21170 (8)	0.0252 (3)	
H16	−0.032413	0.449058	0.223398	0.030*	
C7	0.35190 (10)	0.89704 (16)	0.59075 (9)	0.0276 (3)	
H7	0.349045	0.879951	0.650404	0.033*	
C4	0.14281 (10)	0.32289 (15)	0.64586 (9)	0.0247 (3)	
H4	0.084878	0.287896	0.662230	0.030*	
C5	0.23998 (10)	0.30723 (16)	0.70673 (8)	0.0260 (3)	
H5	0.248753	0.260922	0.764550	0.031*	
C18	0.07681 (11)	0.67486 (17)	0.10673 (9)	0.0306 (3)	
H18	0.068938	0.710281	0.046843	0.037*	
C19	0.16044 (10)	0.72510 (17)	0.17695 (9)	0.0292 (3)	
H19	0.208369	0.798530	0.165462	0.035*	
C10	0.36395 (10)	0.94990 (16)	0.41689 (9)	0.0280 (3)	
H10	0.368583	0.969798	0.358024	0.034*	
C17	0.00479 (10)	0.57280 (17)	0.12428 (9)	0.0307 (3)	
H17	−0.053439	0.540529	0.076612	0.037*	
C11	0.30336 (11)	1.04897 (16)	0.45224 (10)	0.0322 (3)	
H11	0.265829	1.135175	0.416894	0.039*	
C12	0.29735 (11)	1.02292 (16)	0.53841 (10)	0.0334 (3)	
H12	0.255793	1.091263	0.561936	0.040*	
H1	0.4039 (13)	0.447 (2)	0.5188 (12)	0.036 (4)*	

### N-{2-[(E)-2-Phenylethenyl]phenyl}benzenesulfonamide Atomic displacement parameters (Å^2^)

**Table:**

	*U*^11^	*U*^22^	*U*^33^	*U*^12^	*U*^13^	*U*^23^
S1	0.01609 (15)	0.02539 (16)	0.01903 (16)	−0.00204 (10)	0.00389 (11)	−0.00271 (10)
O1	0.0159 (4)	0.0348 (5)	0.0244 (4)	−0.0009 (3)	0.0046 (3)	−0.0016 (4)
O2	0.0253 (4)	0.0339 (5)	0.0197 (4)	−0.0052 (4)	0.0052 (3)	−0.0049 (3)
N1	0.0165 (4)	0.0227 (5)	0.0189 (5)	0.0000 (4)	0.0046 (4)	−0.0018 (4)
C1	0.0176 (5)	0.0178 (5)	0.0212 (5)	0.0009 (4)	0.0064 (4)	−0.0017 (4)
C2	0.0184 (5)	0.0164 (5)	0.0198 (5)	0.0008 (4)	0.0056 (4)	−0.0016 (4)
C6	0.0235 (6)	0.0246 (6)	0.0210 (6)	0.0016 (4)	0.0044 (5)	0.0005 (4)
C3	0.0188 (5)	0.0216 (5)	0.0234 (6)	−0.0002 (4)	0.0069 (4)	−0.0017 (4)
C15	0.0196 (5)	0.0220 (6)	0.0211 (5)	0.0029 (4)	0.0051 (4)	−0.0006 (4)
C13	0.0177 (5)	0.0196 (5)	0.0223 (6)	0.0002 (4)	0.0067 (4)	0.0014 (4)
C14	0.0199 (5)	0.0217 (5)	0.0226 (6)	−0.0019 (4)	0.0066 (4)	0.0001 (4)
C8	0.0180 (5)	0.0203 (5)	0.0261 (6)	−0.0044 (4)	0.0054 (4)	−0.0032 (4)
C9	0.0218 (6)	0.0247 (6)	0.0268 (6)	−0.0023 (5)	0.0077 (5)	−0.0031 (5)
C20	0.0229 (6)	0.0285 (6)	0.0249 (6)	0.0001 (5)	0.0048 (5)	0.0036 (5)
C16	0.0223 (6)	0.0266 (6)	0.0248 (6)	0.0016 (5)	0.0039 (5)	−0.0026 (5)
C7	0.0293 (6)	0.0239 (6)	0.0326 (7)	−0.0036 (5)	0.0139 (5)	−0.0055 (5)
C4	0.0261 (6)	0.0247 (6)	0.0273 (6)	−0.0028 (5)	0.0142 (5)	−0.0015 (5)
C5	0.0322 (6)	0.0267 (6)	0.0203 (6)	−0.0001 (5)	0.0096 (5)	0.0028 (5)
C18	0.0362 (7)	0.0348 (7)	0.0214 (6)	0.0149 (6)	0.0093 (5)	0.0055 (5)
C19	0.0299 (6)	0.0305 (7)	0.0298 (7)	0.0063 (5)	0.0125 (5)	0.0080 (5)
C10	0.0286 (6)	0.0256 (6)	0.0291 (6)	−0.0039 (5)	0.0075 (5)	0.0013 (5)
C17	0.0303 (7)	0.0348 (7)	0.0223 (6)	0.0084 (5)	0.0009 (5)	−0.0032 (5)
C11	0.0329 (7)	0.0210 (6)	0.0422 (8)	0.0004 (5)	0.0106 (6)	0.0022 (5)
C12	0.0367 (7)	0.0221 (6)	0.0456 (8)	0.0022 (5)	0.0189 (6)	−0.0043 (6)

### N-{2-[(E)-2-Phenylethenyl]phenyl}benzenesulfonamide Geometric parameters (Å, º)

**Table:**

S1—O1	1.4422 (9)	C9—H9	0.9500
S1—O2	1.4300 (9)	C9—C10	1.3865 (18)
S1—N1	1.6342 (10)	C20—H20	0.9500
S1—C8	1.7653 (12)	C20—C19	1.3842 (18)
N1—C1	1.4343 (14)	C16—H16	0.9500
N1—H1	0.888 (18)	C16—C17	1.3924 (18)
C1—C2	1.4080 (15)	C7—H7	0.9500
C1—C6	1.3918 (16)	C7—C12	1.391 (2)
C2—C3	1.4061 (16)	C4—H4	0.9500
C2—C13	1.4701 (15)	C4—C5	1.3901 (18)
C6—H6	0.9500	C5—H5	0.9500
C6—C5	1.3894 (17)	C18—H18	0.9500
C3—H3	0.9500	C18—C19	1.388 (2)
C3—C4	1.3859 (17)	C18—C17	1.387 (2)
C15—C14	1.4702 (16)	C19—H19	0.9500
C15—C20	1.4013 (17)	C10—H10	0.9500
C15—C16	1.3993 (16)	C10—C11	1.3916 (19)
C13—H13	0.9500	C17—H17	0.9500
C13—C14	1.3385 (17)	C11—H11	0.9500
C14—H14	0.9500	C11—C12	1.383 (2)
C8—C9	1.3963 (17)	C12—H12	0.9500
C8—C7	1.3900 (17)		
			
O1—S1—N1	105.29 (5)	C10—C9—H9	120.5
O1—S1—C8	107.63 (6)	C15—C20—H20	119.5
O2—S1—O1	119.83 (5)	C19—C20—C15	120.97 (12)
O2—S1—N1	107.85 (5)	C19—C20—H20	119.5
O2—S1—C8	108.68 (6)	C15—C16—H16	119.6
N1—S1—C8	106.87 (5)	C17—C16—C15	120.79 (12)
S1—N1—H1	111.2 (11)	C17—C16—H16	119.6
C1—N1—S1	121.88 (8)	C8—C7—H7	120.6
C1—N1—H1	119.1 (11)	C8—C7—C12	118.90 (13)
C2—C1—N1	120.96 (10)	C12—C7—H7	120.6
C6—C1—N1	117.52 (10)	C3—C4—H4	120.0
C6—C1—C2	121.33 (11)	C3—C4—C5	120.09 (11)
C1—C2—C13	121.39 (10)	C5—C4—H4	120.0
C3—C2—C1	116.96 (10)	C6—C5—C4	119.53 (11)
C3—C2—C13	121.65 (10)	C6—C5—H5	120.2
C1—C6—H6	119.9	C4—C5—H5	120.2
C5—C6—C1	120.27 (11)	C19—C18—H18	120.1
C5—C6—H6	119.9	C17—C18—H18	120.1
C2—C3—H3	119.1	C17—C18—C19	119.80 (12)
C4—C3—C2	121.82 (11)	C20—C19—C18	120.18 (13)
C4—C3—H3	119.1	C20—C19—H19	119.9
C20—C15—C14	122.56 (11)	C18—C19—H19	119.9
C16—C15—C14	119.30 (11)	C9—C10—H10	120.0
C16—C15—C20	118.14 (11)	C9—C10—C11	120.03 (12)
C2—C13—H13	117.3	C11—C10—H10	120.0
C14—C13—C2	125.34 (11)	C16—C17—H17	120.0
C14—C13—H13	117.3	C18—C17—C16	120.06 (12)
C15—C14—H14	117.1	C18—C17—H17	120.0
C13—C14—C15	125.78 (11)	C10—C11—H11	119.7
C13—C14—H14	117.1	C12—C11—C10	120.53 (13)
C9—C8—S1	119.56 (9)	C12—C11—H11	119.7
C7—C8—S1	119.01 (10)	C7—C12—H12	119.9
C7—C8—C9	121.31 (12)	C11—C12—C7	120.25 (13)
C8—C9—H9	120.5	C11—C12—H12	119.9
C10—C9—C8	118.96 (12)		
			
S1—N1—C1—C2	110.23 (11)	C6—C1—C2—C13	−178.89 (11)
S1—N1—C1—C6	−74.82 (13)	C3—C2—C13—C14	−18.42 (18)
S1—C8—C9—C10	175.66 (9)	C3—C4—C5—C6	0.44 (19)
S1—C8—C7—C12	−174.65 (10)	C15—C20—C19—C18	−0.9 (2)
O1—S1—N1—C1	164.82 (9)	C15—C16—C17—C18	−0.97 (19)
O1—S1—C8—C9	37.47 (11)	C13—C2—C3—C4	179.40 (11)
O1—S1—C8—C7	−146.36 (10)	C14—C15—C20—C19	177.62 (12)
O2—S1—N1—C1	35.79 (10)	C14—C15—C16—C17	−176.72 (11)
O2—S1—C8—C9	168.65 (9)	C8—S1—N1—C1	−80.91 (10)
O2—S1—C8—C7	−15.19 (11)	C8—C9—C10—C11	−0.86 (18)
N1—S1—C8—C9	−75.20 (10)	C8—C7—C12—C11	−1.2 (2)
N1—S1—C8—C7	100.96 (10)	C9—C8—C7—C12	1.44 (18)
N1—C1—C2—C3	175.13 (10)	C9—C10—C11—C12	1.1 (2)
N1—C1—C2—C13	−4.13 (16)	C20—C15—C14—C13	−6.09 (19)
N1—C1—C6—C5	−175.42 (11)	C20—C15—C16—C17	2.61 (18)
C1—C2—C3—C4	0.15 (17)	C16—C15—C14—C13	173.20 (12)
C1—C2—C13—C14	160.81 (12)	C16—C15—C20—C19	−1.68 (19)
C1—C6—C5—C4	0.07 (19)	C7—C8—C9—C10	−0.41 (18)
C2—C1—C6—C5	−0.48 (18)	C19—C18—C17—C16	−1.7 (2)
C2—C3—C4—C5	−0.56 (18)	C10—C11—C12—C7	0.0 (2)
C2—C13—C14—C15	−179.52 (11)	C17—C18—C19—C20	2.6 (2)
C6—C1—C2—C3	0.37 (16)		

### N-{2-[(E)-2-Phenylethenyl]phenyl}benzenesulfonamide Hydrogen-bond geometry (Å, º)

*Cg*1 is the centroid of the C1–C6 ring.

**Table:**

*D*—H···*A*	*D*—H	H···*A*	*D*···*A*	*D*—H···*A*
N1—H1···O1^i^	0.888 (18)	2.010 (18)	2.8907 (14)	170.8 (17)
C6—H6···O2^ii^	0.95	2.53	3.3332 (16)	143
C7—H7···O2	0.95	2.54	2.9208 (16)	104
C12—H12···*Cg*1^iii^	0.95	2.59	3.4333 (15)	148
C19—H19···*Cg*1^iv^	0.95	2.81	3.5747 (15)	141

Symmetry codes: (i) −*x*+1, −*y*+1, −*z*+1; (ii) −*x*+1, *y*−1/2, −*z*+3/2; (iii) *x*, *y*+1, *z*; (iv) *x*, −*y*+3/2, *z*−1/2.
